# Anisotropic Janus SiP_2_ Monolayer as a Photocatalyst for Water Splitting

**DOI:** 10.1021/acs.jpclett.0c03841

**Published:** 2021-03-04

**Authors:** Tong Yu, Cong Wang, Xu Yan, Guochun Yang, Udo Schwingenschlögl

**Affiliations:** † State Key Laboratory of Metastable Materials Science & Technology and Key Laboratory for Microstructural Material Physics of Hebei Province, School of Science, Yanshan University, Qinhuangdao 066004, China; ‡ Centre for Advanced Optoelectronic Functional Materials Research and Key Laboratory for UV Light-Emitting Materials and Technology of Ministry of Education, Northeast Normal University, Changchun 130024, China; § Physical Science and Engineering Division (PSE), King Abdullah University of Science and Technology (KAUST), Thuwal 23955-6900, Saudi Arabia

## Abstract

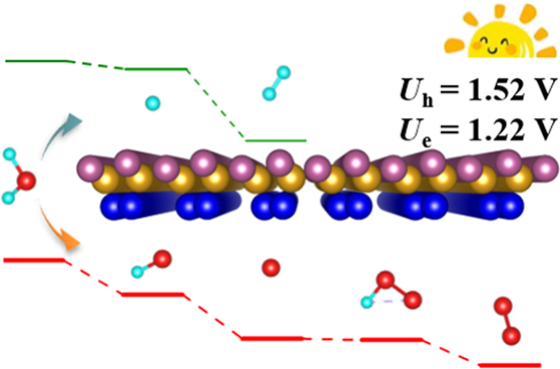

The design of materials meeting the rigorous requirements of photocatalytic water splitting is still a challenge. Anisotropic Janus 2D materials exhibit great potential due to outstandingly high photocatalytic efficiency. Unfortunately, these materials are scarce. By means of ab initio swarm-intelligence search calculations, we identify a SiP_2_ monolayer with Janus structure (i.e., out-of-plane asymmetry). The material turns out to be semiconducting with an indirect band gap of 2.39 eV enclosing the redox potentials of water. Notably, the oxygen and hydrogen evolution half reactions can happen simultaneously at the Si and P atoms, respectively, driven merely by the radiation-induced electrons and holes. The carrier mobility is found to be anisotropic and high, up to 10^–4^ cm^2^ V^–1^ s^–1^, facilitating fast transport of the photogenerated carriers. The SiP_2_ monolayer shows remarkably strong optical absorption in the visible-to-ultraviolet range of the solar spectrum, ensuring efficient utilization of the solar energy.

The modern economy and society demand huge amounts of energy, while the reserves of traditional fossil energy are limited, and the utilization of fossil energy pollutes the environment. Environmentally friendly, low-cost, and sustainable energy sources thus are in urgent demand. Photocatalytic decomposition of water into hydrogen (H_2_) and oxygen (O_2_) is the basis of one of the most promising energy sources, as it directly utilizes clean, renewable, and cost-free solar energy. While several breakthroughs have emerged since the pioneering work of Fujishima and Honda,^1^ the availability of nontoxic and highly efficient catalysts remains a key issue for large-scale applications.^2,3^

2D materials demonstrate unique advantages over traditional bulk materials for achieving highly efficient photocatalysis.^4^ In particular, large surface-to-volume ratios give rise to abundant active sites.^5^ Fast carrier transport and short distances maximize the utilization of the photogenerated carriers,^6^ and dependence of the electron properties on quantities such as the thickness, surface functionalization, and external strain makes it possible to enhance the utilization of the sunlight.^7^

A variety of 2D photocatalytic materials already have been studied experimentally and/or theoretically, such as g-C_3_N_4_,^8^ BN,^9^ phosphorene,^10^ transition-metal dichalcogenides,^11^ PdSeO_3_,^12,13^ and covalent organic frameworks,^14^ some demonstrating excellent efficiency. Still, photocatalysts for water splitting are rare. Thus, besides improving the performance of the known 2D materials,^15^ it is crucial to search for new candidates, not only to elevate the material properties but also to broaden the knowledge of 2D materials in general.^16^

Janus materials, a special kind of 2D materials, draw attention due to their out-of-plane asymmetry, inducing anisotropy, electric polarization, piezoelectricity, and magnetism suitable for novel electronic devices. The Janus structure also is able to improve the utilization of photogenerated carriers.^17^ The prototypical example is MoSSe (obtained by replacement of the S atoms on one side of 2D MoS_2_ with Se atoms), which exhibits large piezoelectricity.^18^ Several Janus materials, particularly M_2_X_3_ (M = Al, Ga, In; X = S, Se, Te)^6^ and B_2_P_6_,^19^ achieve an outstanding photocatalytic efficiency, even in excess of the conventional theoretical limit of 18%. They provide a route to realizing the oxygen evolution reaction (OER) and hydrogen evolution reaction (HER) simultaneously at different atomic species.^20^

Silicene, the 2D counterpart of widely used silicon, has a buckled honeycomb structure, realizes a mixture of sp^2^ and sp^3^ hybridization, and is nonmetallic, in sharp contract to graphene.^21^ Phosphorene combines a direct band gap with anisotropic mechanical, electronic (sp^3^ hybridization), and optical properties originating from its structural asymmetry.^22,23^ Three-coordination of Si or P atoms also facilitates the formation of a 2D structure. Indeed, several stable 2D Si_*x*_P_*y*_ materials have been reported with novel structures and extraordinary properties.^24−26^ While g-C_3_N_4_ shares with phosphorene the excellent catalytic performance, the weak absorption of sunlight limits applications.^27^ However, though C and Si as well as N and P belong to the same group of the periodic table, they are distinguished in terms of their electronegativity and will hybridize differently; i.e., a new 2D Si_*x*_P_*y*_ material still can realize strong optical absorption.

As currently ab initio structural prediction plays an important role in the discovery of new materials,^28−30^ we conduct in the present work a global search for the lowest-energy structure of 2D Si_*x*_P_*y*_ (*x* = 1–4 and *y* = 1–4). We identify stable semiconducting SiP_2_ and SiP_3_ monolayers. Interestingly, the SiP_2_ monolayer realizes an anisotropic Janus structure. It combines high carrier mobilities with strong optical absorption. The Si and P atoms give rise to active sites for the OER and HER, respectively, and it turns out that the photogenerated electrons and holes can trigger the two half reactions to occur simultaneously. This opens great potential of the SiP_2_ monolayer in photocatalytic water splitting.

We apply the crystal structure analysis by particle swarm optimization (CALYPSO) code;^31,32^ see details in the Supporting Information. Structure optimizations and electronic property calculations are performed in the framework of density functional theory, using the Vienna ab initio simulation package^33,34^ and projector augmented-wave^35^ pseudopotentials with Si 3s^2^3p^2^ and P 3s^2^3p^3^ valence states. The energy cutoff of the plane waves is set to 400 eV, the energy convergence to 10^–6^ eV, and the atomic force convergence to 10^–3^ eV Å^–1^. To create 2D models, a vacuum slab of ∼20 Å thickness is adopted. The Perdew–Burke–Ernzerhof^36^ functional is used for the structure optimizations, and to determine accurate band gaps and optical properties, we adopt the Heyd–Scuseria–Ernzerhof (HSE06)^37^ hybrid functional. Deformation potential theory is employed to predict the carrier mobilities;^38^ phonon dispersions are derived by the supercell approach of the Phonopy code,^39^ and molecular dynamics (MD) simulations are executed to evaluate the thermal stability. The MD simulations last 10 ps with a time step of 1 fs and are based on an NVT ensemble with Nosé–Hoover temperature control.^40^ 20 O_2_ molecules are evenly distributed on the two sides of the SiP_2_ monolayer with the distance to the monolayer between 2 and 3 Å.

By extensive structural search, two hitherto unknown 2D materials with stoichiometries of SiP_2_ and SiP_3_ are identified (structural information in Tables S1 and S2). Other stoichiometries are incompatible with dynamical stability. SiP_2_ exhibits out-of-plane asymmetry (Janus structure; Figure 1a,b), consisting of a buckled honeycomb structure with alternating Si and P atoms, like silicene;^41^ and zigzag P chains, like 3D boron monophosphide^42^ and 2D AsP.^43^ Since the P atoms in the zigzag chains are connected to Si atoms, each Si/P atom in the honeycomb structure is three-coordinated with P/Si atoms, and each P atom in the zigzag chains connects to one Si and two P atoms, giving rise to an sp^3^ hybridization and satisfying the chemical octet rule for both the Si and P atoms. Notice that one of the P hybrid orbitals holds an electron lone pair (Figure 1c) and that the unique structural arrangement exposes the P atoms. The bonding is strongly covalent (Figure 1c,d), and the Si–P (2.28 Å) and P–P (2.27 Å) bond lengths are comparable to those in SiP (2.33 Å)^44^ and phosphorene (2.24 Å).^45^

**Figure 1:**
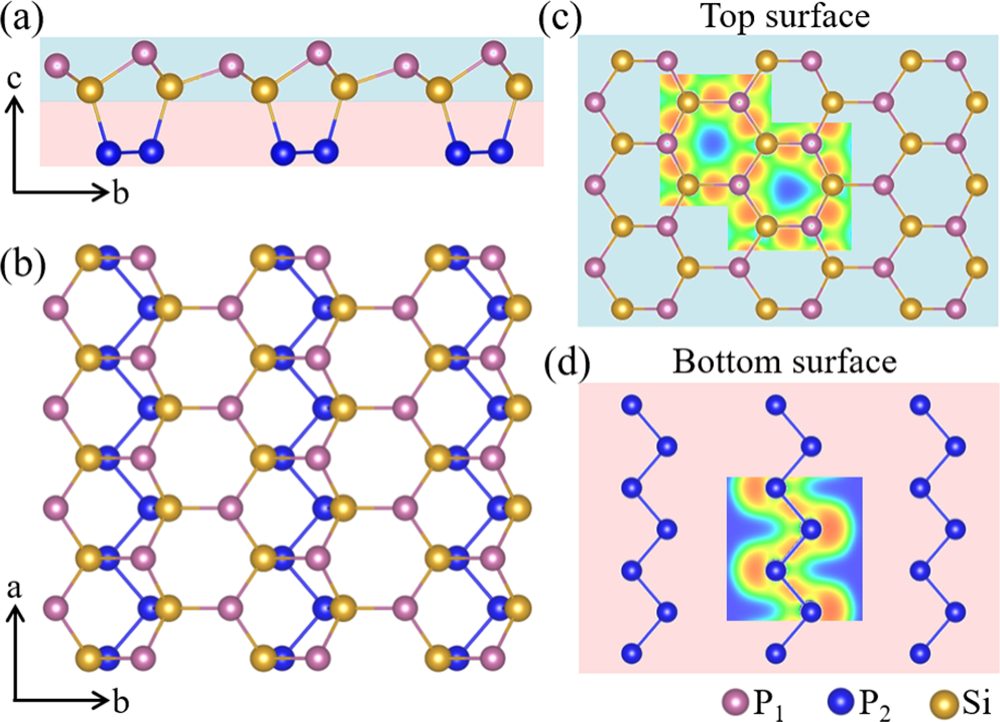
(a) Side and (b) top views of the SiP_2_ monolayer. Electron localization function in the (c) top and (d) bottom surfaces.

Being a prerequisite for application, we explore the dynamical, mechanical, thermal, and air stabilities of the SiP_2_ monolayer. The obtained phonon spectrum is indicative of dynamical stability (absence of imaginary frequencies throughout the Brillouin zone; Figure 2a). The highest frequency (529 cm^–1^) is comparable to results for Si_3_P (540 cm^–1^)^26^ and phosphorene (470 cm^–1^),^46^ demonstrating the formation of strong covalent bonds. MD simulations carried out for 10 ps at 300 and 1000 K show neither bond breaking nor significant structural distortions, verifying thermal stability (Figure S2). As P can easily react with the oxygen (O_2_) molecules in the air, like phosphorene,^47^ and considering that the P atoms of the SiP_2_ monolayer are strongly exposed to the environment, we employ MD simulations at 300 K to check the stability of a SiP_2_ monolayer with 20 O_2_ molecules in a 6 × 3 × 1 supercell. After 10 ps, the SiP_2_ monolayer remains intact, and the O_2_ molecules tend to separate from the monolayer without dissociating into oxygen atoms (Figure 2b). Similar results are obtained for CO_2_, H_2_, N_2_, and H_2_O molecules (Figure S3).

**Figure 2:**
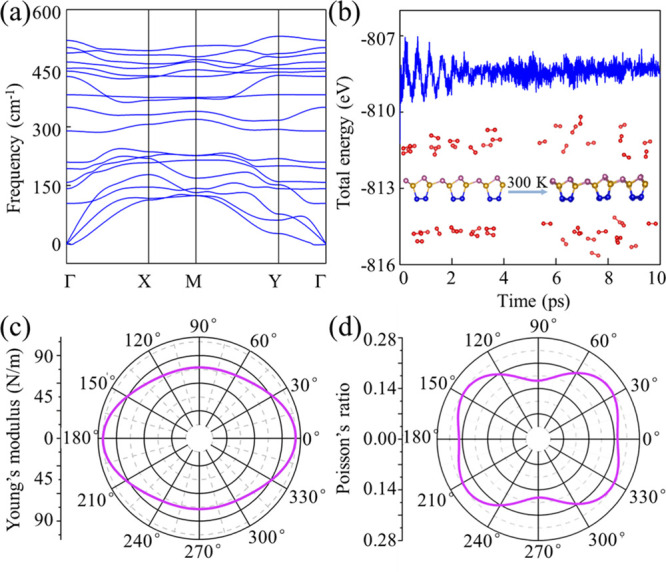
(a) Phonon spectrum of the SiP_2_ monolayer. Phonon densities of states can be found in Figure S1. (b) Total energy and snapshots of the SiP_2_ monolayer with 20 O_2_ molecules before and after a 10 ps MD simulation at 300 K. Polar diagrams of (c) *E*(θ) and (d) *v*(θ).

Based on the calculated linear elastic constants, the SiP_2_ monolayer is also mechanically stable (Supporting Information). Young’s modulus *E*(θ) characterizes a material’s flexibility/stiffness, and Poisson’s ratio *v*(θ) describes its mechanical response to an external load. We find that *E*(θ) varies from 77 to 105 N m^–1^ (Figure 2c), thus being smaller than that of graphene (342 N m^–1^)^48^ but comparable to that of phosphorene (24–102 N m^–1^).^49^ The in-plane flexibility of the SiP_2_ monolayer consequently is moderate. We further find that *v*(θ) varies from 0.16 to 0.24 (Figure 2d). The cohesive energy is useful to evaluate the prospects for experimental synthesis of a predicted 2D material. We find for the SiP_2_ monolayer a value of 3.98 eV atom^–1^. While this value is lower than those reported for graphene (7.91 eV atom^–1^)^50^ and 2D MoS_2_ (5.15 eV atom^–1^),^51^ it surpasses the cohesive energies of already existing silicene (3.91 eV atom^–1^),^19^ germanene (3.24 eV atom^–1^),^19^ and phosphorene (3.30 eV atom^–1^),^52^ indicating feasibility of experimental synthesis of the SiP_2_ monolayer.

We next explore the electronic properties of the SiP_2_ monolayer by studying the electron band structure and partial densities of states (PDOS). At the HSE06 level of theory, we find a semiconducting character with an indirect band gap of 2.39 eV (Figure 3a). Moreover, the conduction band minimum (CBM) is located at the M (0.5, 0.5, 0.0) point, and the valence band maximum (VBM) is located between the M and Y (0.0, 0.5, 0.0) points. Notably, the direct band gap of 2.51 eV at the Y point comes close to the indirect band gap. The PDOS shows strong Si–P hybridization (Figure 3b), pointing to covalent bonds, which is consistent with our above structural analysis. Both the VBM and CBM mainly originate from the Si 3p and P 3p orbitals. The charge densities at the VBM (Figure 3c) and CBM (Figure 3d) indicate that the π electron cloud is broken up by the electron lone pairs, as observed in PC_6_.^53^

**Figure 3:**
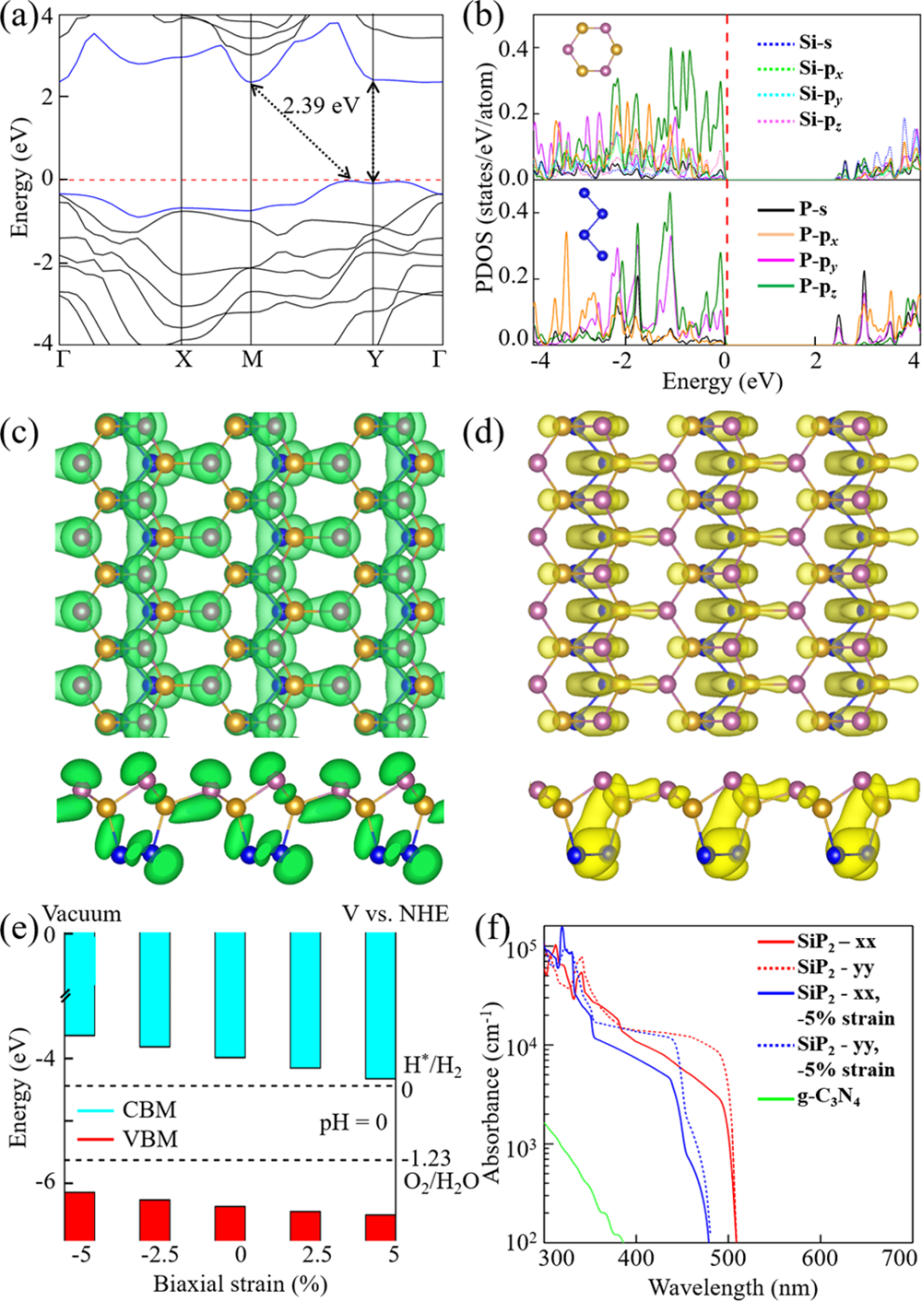
(a) Electronic band structure of the SiP_2_ monolayer. The horizontal dashed line is the VBM. (b) PDOS of the P and Si atoms in the Si_3_P_3_ honeycombs and the P atoms in the zigzag P chains. Top and side views of the charge densities at the (c) VBM and (d) CBM. (e) Energetic positions of the VBM and CBM under biaxial strain. The dashed lines mark the redox potentials of water at pH = 0. (f) Optical absorption coefficient of the SiP_2_ monolayer compared to g-C_3_N_4_.

While, in general, every semiconductor with a band gap between 1.23 and 3 eV is a potential photocatalyst for water splitting, the energetic positions of the VBM and CBM must be significantly lower and higher than the water oxidation potential (−5.67 eV) and hydrogen reduction potential (−4.44 eV) at pH = 0, respectively. The larger the energy differences, the better it is for the water splitting. The VBM (−6.37 eV) and CBM (−3.98 eV) of the SiP_2_ monolayer satisfy the thermodynamic requirements (Figure 3e). This remains valid under up to ±5% strain. While stretching supports the hydrogen reduction, compression supports the water oxidation.

An excellent photocatalyst must be able to harvest sunlight efficiently, particularly visible and ultraviolet light. To determine the optical absorption coefficient of the SiP_2_ monolayer in a reliable manner, the HSE06 level of theory is employed. Between 300 and 500 nm, we obtain values of up to 10^5^ cm^–1^ (Figure 3f), which is much higher than that reported for g-C_3_N_4_.^54^ The obtained anisotropy of the optical adsorption is not very large. More interestingly, we find a red-shift of the spectrum under strain, supporting the utilization of visible light, while both the anisotropy and high optical absorption coefficient are maintained. Hence, the SiP_2_ monolayer can effectively harvest sunlight, facilitating utilization as a photocatalyst for water splitting.

Rapid transport of the photogenerated electrons and holes to the active sites is crucial for a highly efficient catalysis. High carrier mobility is also a prerequisite of many high-performance electronic devices.^20^ We aim to employ deformation potential theory to estimate the carrier mobility of the SiP_2_ monolayer. To verify that this approach is suitable, we predict the hole mobility of phosphorene as 2533 cm^2^ V^–1^ s^–1^, which is consistent with the reported value of 2200 cm^2^ V^–1^ s^–1^.^55^ The main parameters calculated for the SiP_2_ monolayer are given in Table 1. The high carrier mobilities outperform 2D MoS_2_ (200 cm^2^ V^–1^ s^–1^)^56^ and g-C_3_N_4_ (334 cm^2^ V^–1^ s^–1^),^57^ showing strong anisotropy with the lower electron and hole mobility along the *a* and *b* direction, respectively. This is mainly due to the direction-dependences (Table 1) of the deformation potential constant (larger/smaller in the *a* than the *b* direction for electrons/holes) and the effective mass (smaller in the *a* than the *b* direction for both electrons and holes) resulting from the structural anisotropy inherent to the SiP_2_ monolayer. Importantly, the SiP_2_ monolayer is able to ensure fast transport of the photogenerated electrons and holes to effectively participate in the redox reaction. While it is a challenge to achieve 2D materials that combine a large band gap with high carrier mobility and directional control,^58−60^ the SiP_2_ monolayer meets these requirements and thus is also a promising candidate for high-performance electronic devices.

**Table 1:** SiP_2_ Monolayer: Deformation Potential Constant (*E*_DP_), In-Plane Stiffness (*C*), Effective Mass (m*), Carrier Mobility (μ), and Relaxation Time (τ) along the *a* and *b* Directions at 300 K

carrier type	*E*_DP_ (eV)	*C* (J m^–2^)	m* (*m*_0_)	μ (cm^2^ V^–1^ s^–1^)	τ (ps)
electron (*a*)	12.51	101.28	0.13	212.36	0.02
hole (*a*)	0.31	101.28	0.78	3.20 × 10^4^	15.60
electron (*b*)	0.23	76.99	1.90	3.27 × 10^4^	38.80
hole (*b*)	0.58	76.99	1.03	5.27 × 10^3^	3.39

To study whether the photogenerated electrons and holes can provide enough driving force to trigger the OER and HER, we focus on neutral conditions (pH = 7). The energetic positions of the VBM and CBM still enclose the redox potentials of water. The calculated potentials of the photogenerated electrons and holes are *U*_e_ = 0.87 V and *U*_h_ = 1.52 V, respectively, and the obtained reaction pathways, structures, and Gibbs free energies are illustrated in Figure 4a,b. For the OER and HER, absorption is favorable at the Si and P atoms, respectively, due to the higher electronegativity of P as compared to Si. Coexistence of active sites for both reactions boosts the photocatalytic efficiency by avoiding recombination of photogenerated carriers.^20^

**Figure 4:**
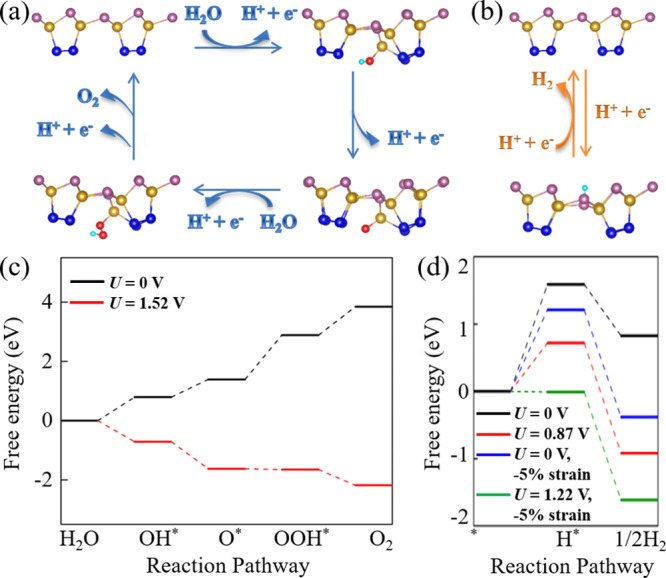
Proposed photocatalytic pathways of the (a) oxygen and (b) hydrogen evolution half reactions on the SiP_2_ monolayer for the (energetically favorable) intermediates OH*, O*, OOH*, and H*. The red and green balls are O and H atoms, respectively. Gibbs free energy diagrams of the (c) OER and (d) HER on the SiP_2_ monolayer for different conditions.

In a dark environment (*U*_h_ = 0 V, black line in Figure 4c), the Gibbs free energy increases in each of the four steps of the OER, indicating that the reaction does not proceed spontaneously. Δ*G*_OOH*_ = 1.50 V is the largest increase of the Gibbs free energy and thus the limiting potential, which consequently is much smaller than in the case of g-C_3_N_4_ (2.28 V).^61^ In a light environment (*U*_h_ = 1.52 V, red line in Figure 4c), the photogenerated holes provide a driving force, and the Gibbs free energy thus decreases in each step; i.e., the molecules can be oxidized into O_2_ in neutral conditions. Like the OER, the HER, which comprises two steps, does not proceed spontaneously in a dark environment (Figure 4d), but it can occur in a light environment under 5% compression. Compression of the SiP_2_ monolayer therefore enhances not only the optical absorption (Figure 3e) but also the driving force of the photogenerated electrons (Figure 3f), enabling efficient photocatalysis.

We finally turn to the discovered SiP_3_ monolayer, which shows a monoclinic structure with space group *C*2/*m* and four formula units per unit cell (Figure 5a–c). The Si and P atoms show sp^3^ hybridization and covalent bonds, satisfying the chemical octet rule. The cohesive energy turns out to be 3.80 eV atom^–1^. It is thus slightly lower than that found for the SiP_2_ monolayer, but exceeds the literature values reported for the SiP (3.64 eV atom^–1^) and Si_3_P (3.78 eV atom^–1^) monolayers.^26^

**Figure 5:**
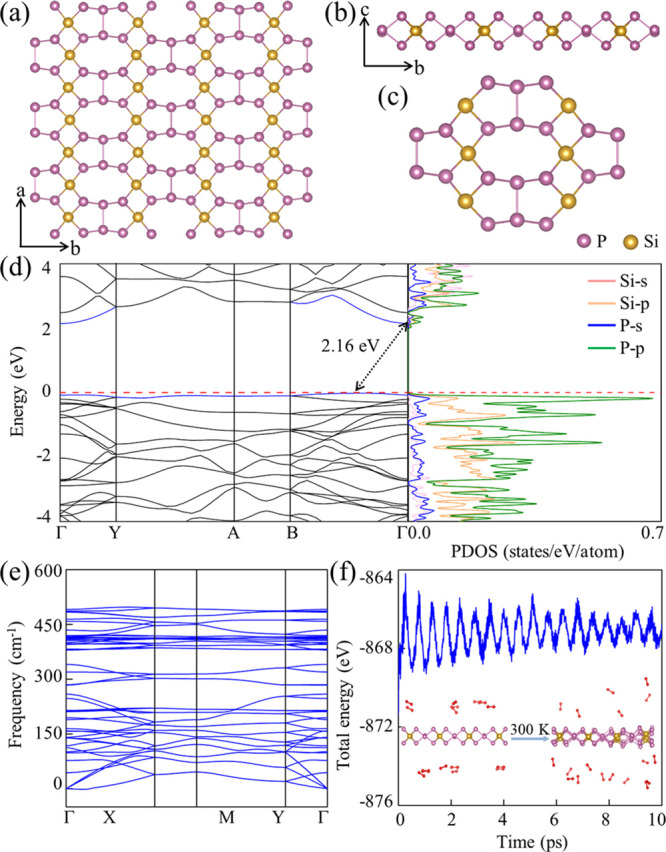
(a) Top and (b) side views of the SiP_3_ monolayer and (c) basic building block (Si_6_P_20_). (d) Electronic band structure and PDOS. The horizontal dashed line is the VBM. (e) Phonon spectrum. Phonon densities of states can be found in Figure S1. (f) Total energy and snapshots of the SiP_3_ monolayer with 20 O_2_ molecules before and after a 10 ps MD simulation at 300 K.

The SiP_3_ monolayer is an indirect band gap (2.16 eV at the HSE06 level of theory; Figure 5d) semiconductor and dynamically (Figure 5e) and thermally (Figure 5f and Figure S2) stable. The electron and hole mobilities are anisotropic, with a particularly large electron mobility along the *a* direction (Table 2). Generally, 2D materials can enhance the performance of field effect transistors due to confinement of the charge carriers in atomically thin channels.^62,63^ Considering its moderate band gap and high electron mobility, outperforming 2D MoS_2_,^56^ the SiP_3_ monolayer has great potential in this field.

**Table 2:** SiP_3_ Monolayer: Deformation Potential Constant (*E*_DP_), In-Plane Stiffness (*C*), Effective Mass (m*), Carrier Mobility (μ), and Relaxation Time (τ) along the *a* and *b* Directions at 300 K

carrier type	*E*_DP_ (eV)	*C* (J m^–2^)	m* (*m*_0_)	μ (cm^2^ V^–1^ s^–1^)	τ (ps)
electron (*a*)	1.16	35.95	0.47	2.33 × 10^3^	0.68
hole (*a*)	1.68	35.95	5.71	12.39	0.04
electron (*b*)	3.01	73.71	0.58	575.38	0.21
hole (*b*)	2.63	73.71	2.58	22.94	0.04

The structural motifs of the SiP_2_ monolayer (zigzag P chains) and SiP_3_ monolayer (Si_6_P_20_ units consisting of edge-sharing Si_2_P_2_ quadrangles and SiP_4_ pentagons) complement the four motifs (Figure S4) reported in the literature for 2D SiP,^25,26,44^ 2D SiP_2_,^24^ and 2D Si_3_P.^26^ In the case of 2D SiP, buckled hexagonal Si_3_P_3_ rings, consisting of alternating Si and P atoms,^26,44^ and interconnected chairlike Si_3_P_3_ and Si_3_P_2_ rings, in which the Si atoms are four-coordinated,^25^ have been reported. In the case of 2D SiP_2_, a chairlike Si_3_P_3_ ring and two Si_2_P_3_ rings share edges, leading to distorted P chains.^24^ The basic structural unit of 2D Si_3_P is Si_6_P_6_, where each Si atom of a central hexagonal ring bonds to a P atom.^26^ Great variability in the structural motifs therefore is found to be characteristic of 2D Si_*x*_P_*y*_.

In conclusion, we discover two 2D materials, the SiP_2_ and SiP_3_ monolayers, through a combination of evolutionary search and ab initio calculations. For the SiP_2_ monolayer we obtain a Janus structure with high thermal and dynamical stabilities resulting from strong Si–P and P–P covalent bonds that satisfy the chemical octet rule. The SiP_2_ monolayer shows remarkably high carrier mobilities of the order of 10^–4^ cm^2^ V^–1^ s^–1^ with preferential electron transport along the *b* direction and hole transport along the *a* direction. Also, the optical absorption coefficient is high. Interestingly, the Si and P atoms give rise to active sites for the OER and HER, respectively, and we find that the material is capable of splitting water into O_2_ and H_2_ under sunlight. Overall, we demonstrate that the SiP_2_ monolayer is an excellent candidate for photocatalytic water splitting.

## Supporting Information Available

Description of the computational methods and detailed structural information on the SiP_2_ and SiP_3_ monolayers (PDF)
