# Supplementary material for: Anisotropic Janus SiP2 Monolayer as a Photocatalyst for Water Splitting
Source: J Phys Chem Lett. 2021 Mar 4;12(9):2464–70. doi: 10.1021/acs.jpclett.0c03841 (PMC8041313; doi:10.1021/acs.jpclett.0c03841)
Supplement: Supplementary file 1 — jz0c03841_si_001.pdf [file jz0c03841_si_001.pdf]

Supporting Information

Anisotropic Janus SiP_2 Monolayer As Photocatalyst for Water Splitting

Tong Yu[1,2†], Cong Wang[1,2†], Xu Yan,[1] Guochun Yang[1,2*], and Udo Schwingenschlögl[3*]

[1]State Key Laboratory of Metastable Materials Science & Technology and Key Laboratory for Microstructural Material Physics of Hebei Province, School of Science, Yanshan University, Qinhuangdao 066004, China

[2]Centre for Advanced Optoelectronic Functional Materials Research and Key Laboratory for UV Light-Emitting Materials and Technology of Ministry of Education, Northeast Normal University, Changchun 130024, China

[3]Physical Science and Engineering Division (PSE), King Abdullah University of Science and Technology (KAUST), Thuwal 23955-6900, Saudi Arabia

**E-mail: yanggc468@nenu.edu.cn*

E-mail: udo.schwingenschlogl@kaust.edu.sa

Index **page**

1. Computational Methods S2

2. Supporting Figures S6

3. Supporting Tables........ S9

4. References S10

Computational Methods

Evolutionary search by the crystal structure analysis by particle swarm optimization (CALYPSO) code[1,2] is employed to find the lowest energy structures of the Si_xP_y (x = 1-4 and y = 1-4) monolayers. Unit cells containing 1, 2, and 4 formula units are considered. In a first step, random structures are constructed with atomic coordinates generated by the crystallographic symmetry operations. Local optimizations are performed by the Vienna ab-initio simulation package (VASP)[3] using the conjugate gradient method until the changes in the Gibbs free energy do not excess 1×10^{-5} eV per cell. Then the 60% of the first generation structures with lowest Gibbs free energy are selected to construct the next generation structures by particle swarm optimization. 40% of the next generation structures are generated randomly. A fingerprinting technique based on bond characterization is applied to strictly avoid identical structures. This procedure significantly enhances the diversity of the structures, which is crucial for the efficiency of the global structure search. In most cases, 1000-1200 structures (20-30 generations) are generated.

Local optimizations and electronic property calculations are performed in the framework of the generalized gradient approximation[4] of density functional theory[5] as implemented in VASP. The Si $3s^2p^2$ and P $3s^2p^3$ atomic orbitals are treated as valence states. To obtain a better energy convergence, we have chosen a plane-wave cutoff energy of 400 eV and Monkhorst-Pack k meshes of $2\pi \times 0.03$ Å^{-1}. The Heyd-Scuseria-Ernzerhof hybrid functional is used to calculate absorption coefficients.

Based on the supercell approach of the Phonopy code,[6] phonon dispersions are calculated for a $6 \times 3 \times 1$ supercell of the SiP_2 monolayer and a $2 \times 1 \times 1$ supercell of the SiP_3 monolayer. Ab-initio molecular dynamics[7] simulations are performed for a $6 \times 3 \times 1$ supercell of the SiP_2 monolayer and a

$4 \times 2 \times 1$ supercell of the SiP_3 monolayer at 300 and 1000 K, controlled by the Nosé-Hoover method, using an *NVT* ensemble for 10 ps with a time step of 1 fs. The cohesive energies of the SiP_2 and SiP_3 monolayers are calculated as

$$E_{coh} = (E_{\mathrm{Si}} + xE_{\mathrm{P}} - E_{\mathrm{SiP}_x})/(1 + x),$$

where E_{Si}, E_{P}, and E_{SiP_x} denote the total energies of a single Si atom, a single P atom, and the SiP_x monolayer ($x = 2, 3$), respectively.

The carrier mobility is estimated by deformation potential theory using as

$$\mu = \frac{\mathrm{e}\hbar^3 C}{k_{\mathrm{B}} T m^* m_{\mathrm{d}} (E_{\mathrm{DP}})^2},$$

where C, E_{DP}, and m^* are the elastic modulus for uniaxial strain, the deformation potential constant (VBM for holes and CBM for electrons), and the carrier effective mass in the transport direction, respectively. C is obtained from $(E–E_0)/S_0 = C(\Delta l_0/l_0)^2/2$, where E, E_0, S_0, l_0, and Δl_0 represent the total energy of the strained structure (Figures S5 and S6), the total energy of the equilibrium structure, the equilibrium lattice area, the equilibrium lattice constant in the transport direction, and the change of l_0, respectively. We have $T = 300$ K, $m_{\mathrm{d}} = \sqrt{m_x^* m_y^*}$, and $E_{\mathrm{DP}} = \Delta V_{\mathrm{i}}/(\Delta l_0/l_0)$, where ΔV_{i} is the energy change of the i-th state under compression/tension. The carrier relaxation time is estimated as $\tau = \mu m^*/\mathrm{e}$.

We obtain for the SiP_2 monolayer (in kbar)

$$C = \begin{bmatrix} 427.22 & 68.95 & -0.32 & 0.05 & 0.60 & -0.03 \\ 68.95 & 314.43 & 0.14 & 0.00 & -0.88 & 0.01 \\ -0.32 & 0.14 & 4.00 & -0.02 & 0.76 & 0.00 \\ 0.05 & 0.00 & -0.02 & 132.73 & -0.05 & -0.42 \\ 0.60 & -0.88 & 0.76 & -0.05 & 0.21 & 0.04 \\ -0.03 & 0.01 & 0.00 & -0.42 & 0.04 & 0.43 \end{bmatrix},$$

which fulfills the necessary and sufficient elastic stability conditions.[8] Diagonalization yields

$$C = \begin{bmatrix} 0.05 & 0 & 0 & 0 & 0 & 0 \\ 0 & 0.43 & 0 & 0 & 0 & 0 \\ 0 & 0 & 4.14 & 0 & 0 & 0 \\ 0 & 0 & 0 & 132.73 & 0 & 0 \\ 0 & 0 & 0 & 0 & 281.75 & 0 \\ 0 & 0 & 0 & 0 & 0 & 459.90 \end{bmatrix}.$$

To evaluate the in-plane stiffness, we derive the direction-dependent (θ = angle to the armchair direction) Young's modulus

$$E(\theta) = \frac{C_{11}C_{12} - C_{12}^2}{C_{11}sin^4\theta + C_{22}cos^4\theta + (\frac{C_{11}C_{12} - C_{12}^2}{C_{44}} - 2C_{12})\cos^2\theta\sin^2\theta}$$

and Poisson's ratio

$$v(\theta) = -\frac{\left(C_{11} + C_{22} - \frac{C_{11}C_{12} - C_{12}^2}{C_{44}}\right)\cos^2\theta\sin^2\theta - C_{12}(\sin^4\theta + \cos^4\theta)}{C_{11}\sin^4\theta + C_{22}\cos^4\theta + (\frac{C_{11}C_{12} - C_{12}^2}{C_{44}} - 2C_{12})\cos^2\theta\sin^2\theta}$$

from the elastic constants.

In aqueous solution, the oxygen evolution reaction generally involves the oxidation steps

$$H_2O + * \rightarrow OH* + H^+ + e^- , \quad (1)$$

$$OH* \rightarrow O* + H^+ + e^- , \quad (2)$$

$$O*+ H_2O \rightarrow OOH* + H^+ + e^- , \quad (3)$$

$$OOH* \rightarrow * + O_2 + e^- , \quad (4)$$

where * denotes the adsorption site and OH*, O*, and OOH* denote the adsorbed intermediates. On the other hand, the hydrogen evolution reaction involves the proton/electron transfer and H_2 release steps

$$* + H^+ + e^- \rightarrow H* , \quad (5)$$

$$H* + H^+ + e^- \rightarrow * + H_2 . \quad (6)$$

Considering the electrode potential (*U*) and pH, the free energy changes of the oxygen and hydrogen evolution reactions can be expressed as

$$\Delta G_1 = G_{OH*} + ½G_{H2} – G_{H2O} – G* – \Delta G_U – \Delta \underline{G}_{pH} , \quad (7)$$

$$\Delta G_2 = G_{O*} + ½G_{H2} – G_{OH*} – \Delta G_U – \Delta G_{pH} , \quad (8)$$

$$\Delta G_3 = G_{OOH*} + ½G_{H2} – G_{H2O} – G_{O*} – \Delta G_U – \Delta G_{pH} , \quad (9)$$

$$\Delta G_4 = G_* + ½G_{H2} + G_{O2} – G_{OOH*} – \Delta G_U – \Delta \underline{G}_{pH} , \quad (10)$$

$$\Delta \mathrm{G}_5 = \mathrm{G}_{H*} – ½G_{H2} – G_* – \Delta G_U + \Delta G_{pH} , \quad (11)$$

$$\Delta G_6 = G_* + ½G_{H2} – G_{H*} – \Delta G_U + \Delta G_{pH} . \quad (12)$$

The potential U_e of the photo-generated electrons for the hydrogen reduction, defined as energy difference between the hydrogen reduction potential and the CBM, is found to be 0.46 V for the SiP_2 monolayer at pH = 0. The potential U_h of the photo-generated holes for the water oxidation, defined as energy difference between the VBM and the hydrogen reduction potential, is found to be 1.93 V for the SiP_2 monolayer at pH = 0. Since U_e and U_h refer to the standard hydrogen electrode, they change with the pH as $U_e = 0.46 + 0.059 \times \mathrm{pH}$ and $U_h = 1.93 - 0.059 \times \mathrm{pH}$.

Supporting Figures

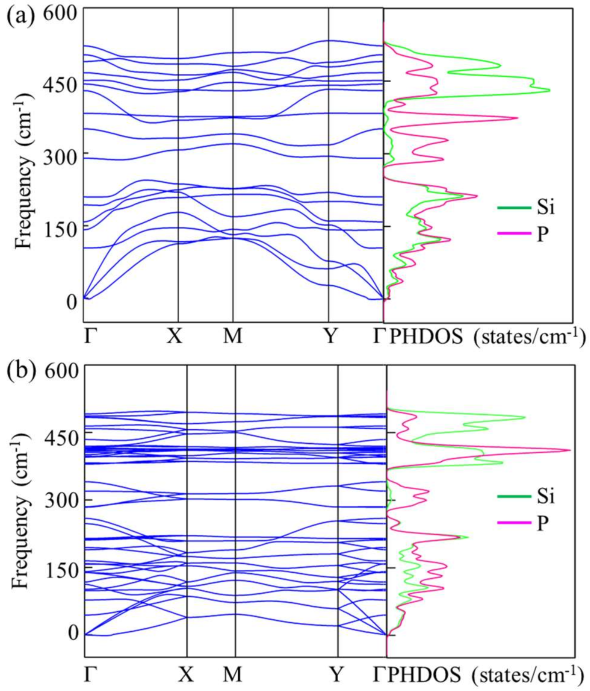

Figure S1. Phonon spectra and partial densities of states of the (a) SiP_2 and (b) SiP_3 monolayers.

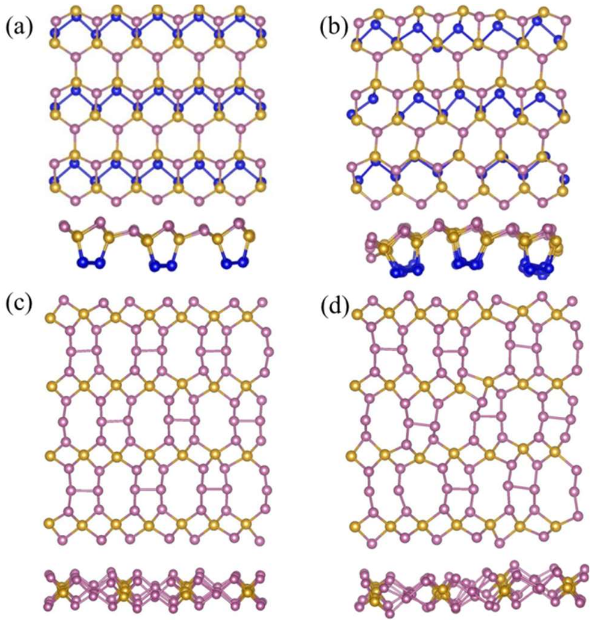

Figure S2. Snapshots of the SiP_2 monolayer after 10 ps MD simulations at (a) 300 K and (b) 1000 K. Snapshots of the SiP_3 monolayer after 10 ps MD simulations at (c) 300 K and (d) 1000 K.

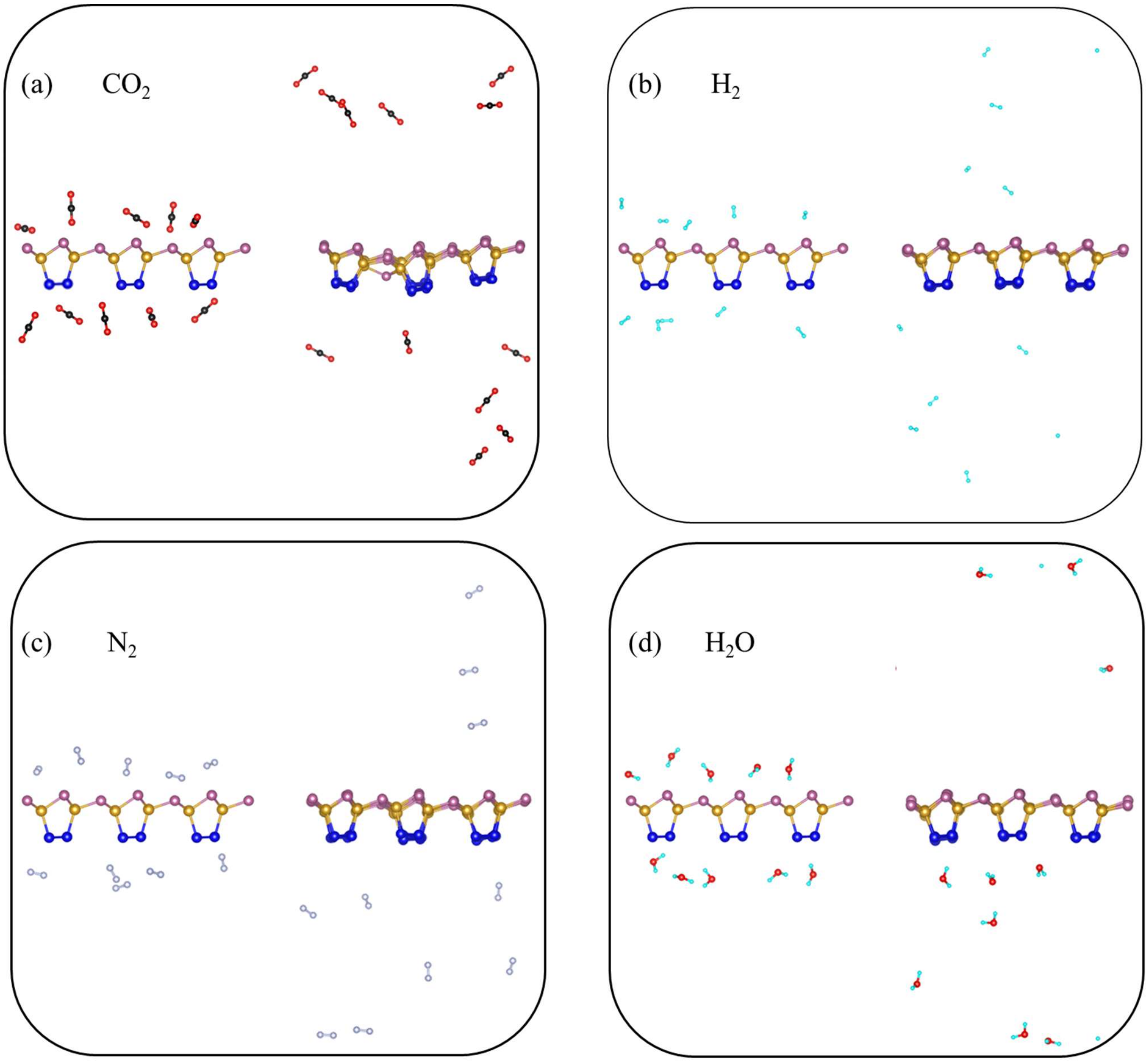

Figure S3. Snapshots of the SiP_2 monolayer with 10 (a) CO_2, (b) H_2, (c) N_2, and (d) H_2O molecules before and after a 5 ps MD simulation at 300 K.

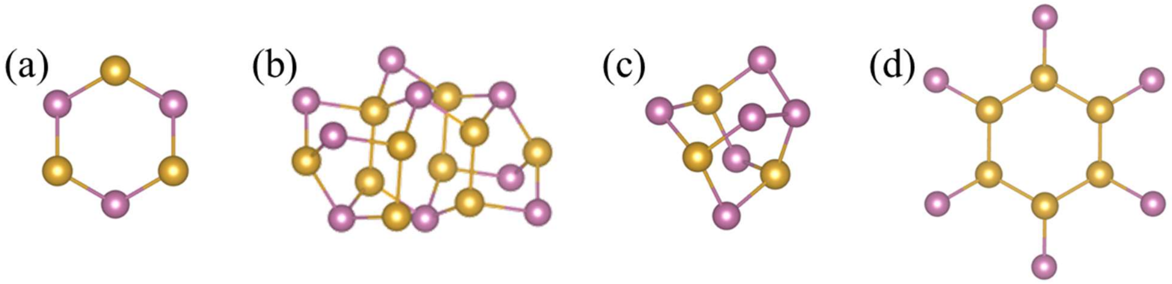

Figure S4. Structural motifs of (a,b) 2D SiP, (c) 2D SiP_2, and (d) 2D Si_3P.

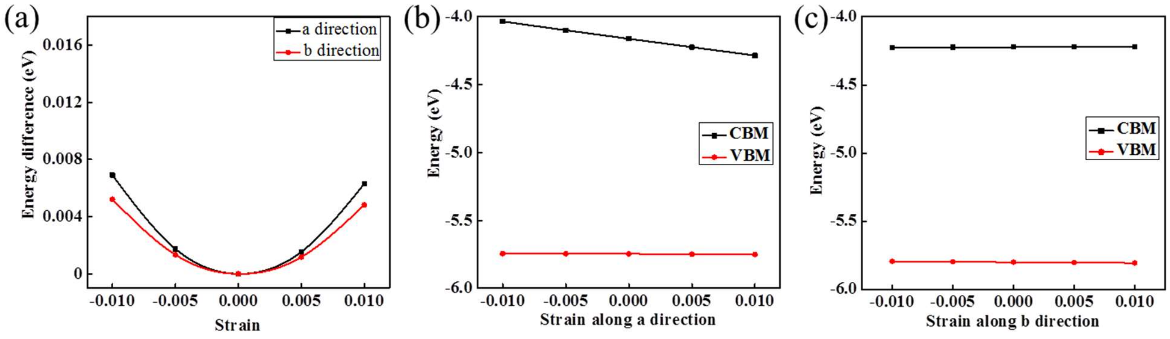

Figure S5. a) Total energy difference between the unstrained and strained (*a* and *b* directions) SiP_2 monolayer. Energy shifts of the VBM and CBM under strain along the (b) *a* and (c) *b* directions.

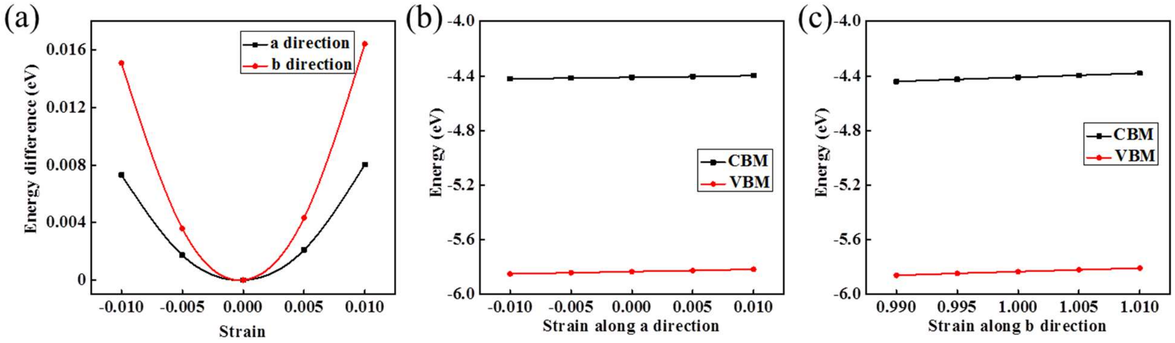

Figure S6. (a) Total energy difference between the unstrained and strained (*a* and *b* directions) SiP_3 monolayer. Energy shifts of the VBM and CBM under strain along the (b) *a* and (c) *b* directions.

Supporting Tables

Table S1. Structural information of the SiP_2 monolayer.

Phase	Space Group	Lattice Parameters (Å, °)	Wyckoff Positions	Fractional Coordinates		
				x	*y*	*z*
SiP_2	*P1*	*a* = 3.454	Si(1a)	0.30878	0.38735	0.51381
		b = 6.045	Si(1a)	0.80912	0.84207	0.51822
		c = 25.412	P(1a)	0.80245	0.72275	0.42973
		α = 92.1	P(1a)	0.30226	0.47913	0.42725
		β = 90.6	P(1a)	0.31259	0.70507	0.56427
		γ = 90.0	P(1a)	0.81126	0.20124	0.54670

Table S2. Structural information of the SiP_3 monolayer.

Phase	Space Group	Lattice Parameters (Å, °)	Wyckoff Positions	Fractional Coordinates		
				x	*y*	*z*
SiP_3	*C2/m*	*a* = 5.714	Si(4h)	0.50000	0.35500	0.43725
		b = 11.967	P(8j)	0.21019	0.25626	0.50000
		c = 20.766	P(4i)	0.30171	0.00000	0.49496
		α = *γ* = 90.0				
		β = 97.6				

References

(1) Wang, Y.; Lv, J.; Zhu, L.; Ma, Y. CALYPSO: A Method for Crystal Structure Prediction. *Comput. Phys. Commun.* **2012**, *183*, 2063–2070.

(2) Wang, Y.; Lv, J.; Zhu, L.; Ma, Y. Crystal Structure Prediction Via Particle-Swarm Optimization. *Phys. Rev. B.* **2010**, *82*, 94116.

(3) Kresse, G.; Furthmüller, J. Efficient Iterative Schemes for Ab Initio Total-Energy Calculations Using a Plane-Wave Basis Set. *Phys. Rev. B.* **1996**, *54*, 11169–11186.

(4) Perdew, J. P.; Burke, K.; Ernzerhof, M. Generalized Gradient Approximation Made Simple. *Phys. Rev. Lett.* **1996**, *77*, 3865–3868.

(5) Kohn, W.; Sham, L. J. Self-Consistent Equations Including Exchange and Correlation Effects. *Phys. Rev.* **1965**, *140*, A1133–A1138.

(6) Togo, A.; Oba, F.; Tanaka, I. First-Principles Calculations of the Ferroelastic Transition between Rutile-Type and $CaCl_2$-Type SiO_2 at High Pressures. *Phys. Rev. B* **2008**, *78*, 134106.

(7) Martyna, G. J.; Klein, M. L.; Tuckerman, M. Nosé-Hoover Chains: The Canonical Ensemble Via Continuous Dynamics. *J. Chem. Phys.* **1992**, *97*, 2635–2643.

(8) Mouhat, F.; Coudert, F.-X. Necessary and Sufficient Elastic Stability Conditions in Various Crystal Systems. *Phys. Rev. B* **2014**, *90*, 224104.
